# Neutrophil-to-Lymphocyte Ratio and Platelet-to-Lymphocyte Ratio in Discriminating Precancerous Pathologies from Cervical Cancer

**DOI:** 10.1155/2019/2476082

**Published:** 2019-08-29

**Authors:** Mustafa Tas, Adem Yavuz, Mehmet Ak, Bulent Ozcelik

**Affiliations:** ^1^Department of Obstetrics and Gynecology, Acıbadem Mehmet Ali Aydınlar University, Acıbadem Kayseri Hospital, Kayseri, Turkey; ^2^Department of Obstetrics and Gynecology, Ömer Halisdemir University, Niğde, Turkey; ^3^Department of Obstetrics and Gynecology, Kayseri City Hospital, Kayseri, Turkey; ^4^Department of Obstetrics and Gynecology, Erciyes University, Kayseri, Turkey

## Abstract

**Purpose:**

We aimed to determine the predictive value of several hematological markers of inflammation on the presence/absence of cervical cancer and also to determine their ability in discriminating precancerous cervical pathologies from cervical cancer.

**Materials and Methods:**

In this study, patients who presented to Acıbadem Kayseri Hospital between May 2010 and June 2018 were evaluated. Forty patients with low-grade squamous intraepithelial lesions (LSIL), 40 patients with high-grade squamous intraepithelial lesions (HSIL), and 30 patients with cervical cancer (CC) were retrospectively included in this study. A control group of 70 healthy volunteers with normal cervical cytology was also included in the study.

**Results:**

The neutrophil-to-lymphocyte ratio (NLR) was significantly higher in patients with CC than in controls. The platelet-to-lymphocyte ratio (PLR) was significantly higher in patients with CC compared to those with LSIL and HSIL diagnoses and also controls (*p* < 0.001). Logistic regression analysis revealed that age (OR: 1.075, 95% CI: 1.020–1.132, *p*=0.007), NLR (OR: 1.643, 95% CI: 1.009–3.142, *p*=0.047), and PLR (OR: 1.032, 95% CI: 1.003–1.062, *p*=0.029) were predictors for the presence of CC. ROC curve analysis revealed that both NLR and PLR were predictive of CC with a cutoff value of 2.02 for NLR (71% sensitivity and 60% specificity, AUC: 0.682, *p*=0.004) and 126.7 for PLR (83% sensitivity and 69% specificity, AUC: 0.752, *p* < 0.001).

**Conclusion:**

In addition to patients' age, determination of NLR and PLR values, which are simple, inexpensive, and readily available markers of systemic inflammation, may help in decision making precancerous pathologies of the cervix.

## 1. Introduction

Globally, cervical cancer (CC) is among the three most common malignancies seen in women and leads to substantial mortality of approximately 250,000 patients each year, particularly in developing countries [1]. Novel screening methods which have been introduced in the last 20 years, such as visual inspection with acetic acid (VIA) and HPV DNA testing, have led to improvements in early detection and treatment of cervical pathologies, either as primary screening tests in combination with cytology or as adjunctive to cytology findings. Despite the advances in novel screening tests, most women in developing countries still present in the advanced stages of the disease, at a time when they are usually untreatable and only palliative measures can be taken [2]. The lack of sufficient population screening and the relatively low sensitivity of present tests necessitate the introduction of simple, inexpensive, and widely available markers as standalone tests for the screening of CC. Furthermore, tests that are capable of increasing the diagnostic accuracy of existing tests may also be critical for early diagnosis and treatment of cervical lesions.

The issue of inflammation has received considerable critical attention in not only initiation and promotion but also in the progression, invasion, and metastasis of a tumor. Recently, several surrogate markers of systemic inflammation such as serum C-reactive protein, neutrophil-to-lymphocyte ratio (NLR), and platelet-to-lymphocyte ratio (PLR) have been found to be associated with various kinds of cancers including non-small-cell lung cancer, pancreatic adenocarcinoma, gastric cancer, over carcinoma, renal cell carcinoma, colorectal cancers, and endometrial cancers [3–7]. Previous data have shown that precancerous pathologies of the cervix carry the potential of developing into CC. For instance, 15% of low-grade squamous intraepithelial lesions (LSILs) and 30% to 45% of high-grade squamous intraepithelial lesions (HSILs) have been reported to progress into severe disease [8]. Therefore, early recognition and discrimination of these lesions are crucial to determine patients who require aggressive treatment.

Limited data derived from previous studies suggest that both NLR and PLR are associated with unfavorable prognosis, lymph node and distant metastasis, and response to radiation therapy in patients with CC [9, 10]. However, the diagnostic ability of inflammatory markers in terms of predicting the presence/absence of precancerous cervical pathologies and CC, and the ability of these markers to discriminate precancerous cervical lesions from cancers have not been investigated yet.

The aim of the present study was to investigate several inflammation markers in terms of CC diagnosis and also their ability to discriminate patients with precancerous cervical lesions from those with CC.

## 2. Materials and Methods

### 2.1. Patient Selection

A total of 110 patients aged between 20 and 65 years and diagnosed with low-grade squamous intraepithelial lesions (LSIL), high-grade squamous intraepithelial lesions (HSIL) or CC in Acıbadem Kayseri Hospital between May 2010 and June 2018 were included in this retrospective study. Data regarding the clinical features, cervical cytology, and pretreatment laboratory tests including white blood cell (WBC) count, hemoglobin, red cell distribution width, plateletcrit (PCT), platelet count, and mean platelet volume (MPV) of all patients were retrieved from hospital records. Patients were divided into three groups according to cervical cytology and histopathological examination results: LSIL, HSIL, and invasive CC (24 with squamous cell carcinoma and 6 with adenocarcinoma). The PLR was calculated by dividing absolute platelet count by absolute lymphocyte count, while the NLR was calculated by dividing absolute neutrophil count by absolute lymphocyte count. In order to eliminate the effects of confounders that might influence laboratory measurements, patients with any hematological disease, cardiovascular disease, previous malignancy, advanced liver or kidney disease, diabetes mellitus, and allergic asthma and those with active infections were not included in the study. The control group was comprised of 70 healthy volunteers with normal cervical cytology. The study was approved by the Institutional Ethical Committee of Acıbadem Mehmet Ali Aydınlar University, and the study was carried out in accordance with the most recent version of the Helsinki Declaration.

### 2.2. Statistical Analysis

Statistical analyses were performed with SPSS software for Windows, version 20 (SPSS, Chicago, IL, USA). Continuous variables were presented as mean + standard deviation, and categorical variables were presented as frequency (*n*) and percentage (%). The Kolmogorov–Smirnov test was used to determine whether or not continuous variables were normally distributed. One-way analysis of variance (ANOVA) and post hoc Tukey tests were used to compare groups. The Pearson correlation coefficient was used to examine the association between selected variables and the presence of cervix cancer. A receiver operating characteristic (ROC) curve was constructed to determine the predictive role of NLR and PLR in identifying precancerous cervical pathologies and CC. Logistic regression analysis was performed to examine the predictive value of age and laboratory measurements on the presence of CC. A multiple linear regression model was used to identify independent predictors of FIGO stage in patients with CC. A two-tailed *p* < 0.05 was considered significant Table 1.

## 3. Results

**Figure 1:**
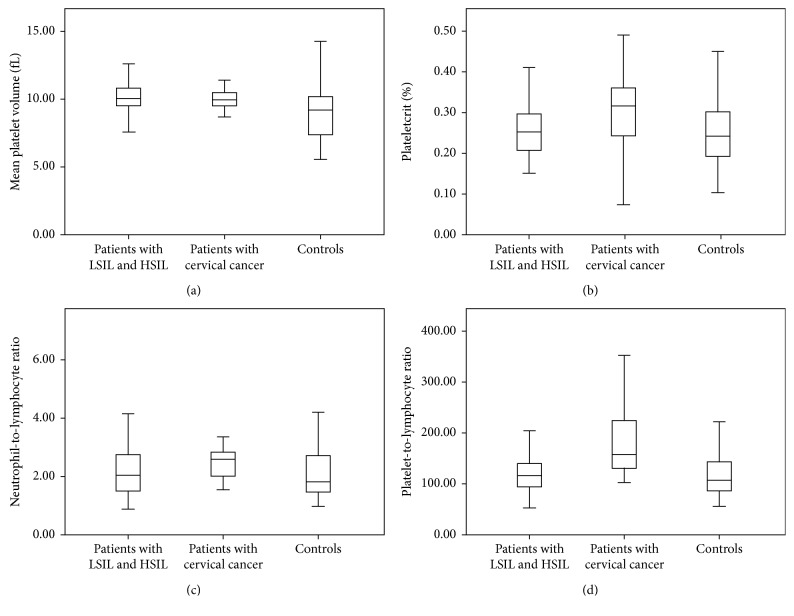
Graphics demonstrating neutrophil-to-lymphocyte ratio, platelet-to-lymphocyte ratio, mean platelet volume, and plateletcrit levels in patients with cervical precancerous pathologies and cervical cancer and controls.

The mean age of the study group was 38 ± 10 years. Comparison of laboratory measurements of patients with LSIL, HSIL, CC, and controls is presented in Table 2. The mean age of patients with CC was higher than that of patients with LSIL and HSIL, as well as controls (46.1 ± 11 years vs. 39.4 ± 9 years, 35.8 ± 7 years, and 35.4 ± 11 years, respectively, *p* < 0.001). The mean hemoglobin level of patients with CC was also lower than that of patients with LSIL (12.2 ± 1.7 g/dL vs. 13.2 ± 1.2 dL, *p*=0.033). The PCT (0.30 ± 0.1% vs. 0.24 ± 0.1%, *p*=0.004) and NLR were significantly higher in patients with CC than in controls but similar to those with LSIL and HSIL. In patients with LSIL, HSIL, or CC, mean MPV was significantly higher compared to controls (9.9 ± 1.1 fL, 10.1 ± 1.0 fL, and 10.0 ± 0.8 fL vs. 8.9 ± 1.6 fL, *p* < 0.05 for all comparisons). The PLR was also significantly higher in patients with CC than in those with LSIL and HSIL and controls (191 ± 83 vs. 123 ± 46, 123 ± 34, and 119 ± 45, *p* < 0.001 for all comparisons, Figure 1).

**Figure 2:**
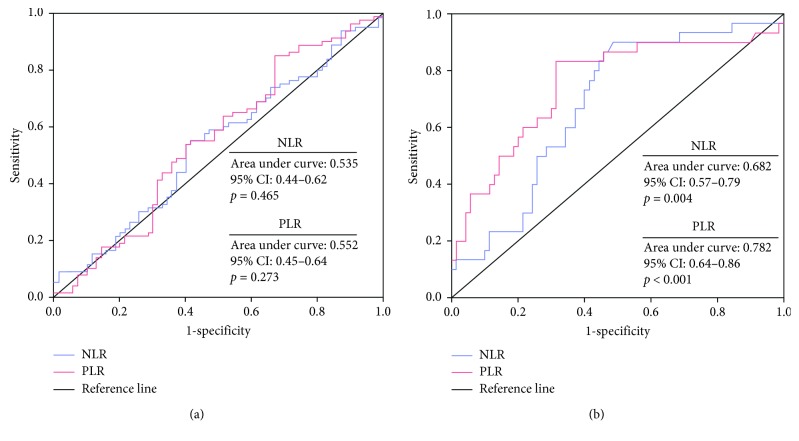
(a) Neutrophil-to-lymphocyte ratio and platelet-to-lymphocyte may predict the presence of cervical cancer with a high sensitivity and specificity. (b) The sensitivity and specificity of neutrophil-to-lymphocyte ratio and platelet-to-lymphocyte ratio are extremely low in predicting cervical precancerous pathologies.

Table 3 shows the results of the correlation analyses carried out to search the association with the presence of CC and selected parameters of complete blood count. Correlation analyses revealed that the presence of CC was significantly correlated with PLR (*r* = 0.452, *p* < 0.001) and NLR (*r* = 0.223, *p*=0.003). Moreover, PLR was significantly correlated with the depth of stromal infiltration (*r* = 0.545, *p*=0.007), tumor size >4 cm (*r* = 0.438, *p*=0.037), and lymph node metastasis (*r* = 0.442, *p*=0.035). ROC curve analysis revealed that both NLR and PLR were predictive of CC with a cutoff value of 2.02 for NLR (71% sensitivity and 60% specificity, AUC: 0.682, *p*=0.004) and 126.7 for PLR (83% sensitivity and 69% specificity, AUC: 0.752, *p* < 0.001, Figure 2). However, neither NLR (AUC: 0.535, 95% CI: 0.44–0.62, *p*=0.4659) nor PLR (AUC: 0.552, 95% CI: 0.45–0.64, *p*=0.273) were predictive for the presence of LSIL and HSIL in ROC curve analysis. The results of logistic regression analysis showed that age (OR: 1.075, 95% CI: 1.020–1.132, *p*=0.007), NLR (OR: 1.643, 95% CI: 1.009–3.142, *p*=0.047), and PLR (OR: 1.032, 95% CI: 1.003–1.062, *p*=0.029) were associated with CC presence (Table 4). In patients with invasive CC, linear regression analysis revealed that NLR (coefficient *β* = 0.315, 95% CI: 0.026–0.605, *p*=0.034) and PLR (coefficient *β* = 0.005, 95% CI: 0.001–0.009, *p*=0.014) were independently associated with the FIGO stage of cancer (Table 5).

## 4. Discussion

The present study demonstrates that patients with invasive CC have higher PLR compared to patients with LSIL and HSIL. Furthermore, our findings indicate that both increased NLR and increased PLR are predictive for the presence of CC. However, PLR and NLR have no value in the prediction of precancerous pathologies of the cervix, including LSIL and HSIL. Moreover, in patients with CC, the NLR and the PLR are independent predictors of the FIGO stage of cancer. We also found increased PCT in patients with invasive CC compared to controls. Nonetheless, PCT has limited value in discriminating invasive CC from LSIL and HSIL.

A strong relationship between NLR and inflammation has been reported in previous studies. Increased neutrophil concentration is accepted to endorse neoplastic progression, and it can repress antineoplastic properties of lymphocytes. Accordingly, NLR may be recognized as the marker of the balance between precancerous inflammatory state and cancerous immune state, and higher NLR might be indicative for tumor development [11]. Results of previous studies suggest that NLR is strongly associated with overall survival in patients with various types of tumors, such as those originating from the lung, liver, and ovary [12–14].

Another representative index of systemic inflammation, PLR, has emerged as an interesting parameter due to the role of inflammation in cancer development and prognosis. A number of studies have demonstrated the prognostic value of PLR in various types of cancer including colorectal cancer, hepatocellular carcinoma, non-small-cell lung cancer, and ovarian cancer [15, 16]. Despite the considerable role of genetic basis in the development of malignancies, a substantial amount of recent data indicate that host immune response is also noteworthy in both the development and progression of cancer. Parameters that show the systemic inflammatory response, such as increased CRP and reduced albumin levels, have been shown to be associated with prognosis in various cancers, particularly in patients with non-small-cell lung cancer, pancreatic adenocarcinoma, gastric cancer, colorectal cancers, and renal cell carcinoma [17].

Plateletcrit (PCT) is identical to the volume that platelets have in 100 milliliters of total blood. PCT constitutes a component of the platelet indices and reflects platelet activity. Hence, high PCT indicates the higher activity of the platelets. Recent data signify that PCT acts as a surrogate marker of the systemic inflammatory response in addition to MPV, NLR, and PLR. However, the debate on the role of PLR in malignancies has not been completed yet as a consequence of the insufficient data investigating PCT in patients with cancer [18]. A short while ago Oncel and colleagues have shown that PCT was increased in patients with metastatic lung cancer compared to nonmetastatic ones [19]. This relationship may partly be explained by the activity of platelets in promoting tumor growth via increasing angiogenesis. Our results for the first time show that PCT is increased in patients with CC compared to controls.

A growing body of evidence highlights the associations between the NLR or PLR and tumor characteristics in patients with CC. Increased NLR and PLR have been shown to be associated with stage, invasiveness, prognosis, and unfavorable histopathological characteristics of CC [20, 21]. Moreover, some of the studies have found that NLR and PLR are associated with LN metastasis and recurrences following chemoradiation therapy in patients with CC [22]. Our results confirm that patients with invasive CC have higher NLR and PLR compared to those with precancerous cervical pathologies. Our findings of significant correlations between PLR value and stromal infiltration depth, tumor size, and lymph node metastasis are consistent with the literature on this topic. In addition, the relationships between the NLR and PLR values and the stage of CC demonstrated in the current study are also in accordance with the findings of previous research. However, none of the studies conducted up to this point have evaluated the discriminative property of NLR and PLR in precancerous and cancerous lesions of the cervix. The present study is the first to evaluate the role of NLR and PLR in differentiating precancerous lesions from invasive CC.

The exact mechanisms underlying these results are unclear. However, there are several possible explanations for this result. Neutrophils and platelets both act in the progression and proliferation of tumor cells by influencing their vascularization. Furthermore, lymphocytes which are crucial in preventing the proliferation and metastasis of tumor cells are decreased with the advancing stages of cancer leading to higher NLRs and PLRs [23]. Our results may also reflect an ongoing immune dysfunction occurring in patients with invasive CC but not in patients with precancerous cervical pathologies. We speculate that destruction in host immune surveillance is somewhat responsible for the initiation and progression of CC. Another possible explanation for our findings is that neutrophils release several cytokines and several phagocytic mediators which are capable of inducing damage to cellular DNA, stimulate angiogenesis, and impede apoptosis [24, 25]. Additionally, platelets release various growth factors, including platelet-derived growth factor and vascular endothelial growth factor (VEGF) which promote vascularization and proliferation of the tumors [26, 27]. Increased NLR and PLR as a result of increased neutrophil and platelet counts may contribute to the stimulation of cancer development in patients with precancerous cervical pathologies.

This study also has some limitations to be mentioned. The present study is a retrospective study and included a relatively small number of patients, especially in terms of the number of patients with invasive CC. These results, therefore, need to be interpreted with caution. In addition, the etiologies of CC cases were not recorded and analyzed; this may have masked the effects of these factors on the inflammatory characteristics of our patients. Finally, the utilization of additional markers of inflammation, such as CRP, procalcitonin, and erythrocyte sedimentation rate, may have provided additional data for the evaluation of our hypothesis; however, these markers were not measured.

In addition to patients' age, determination of NLR and PLR values, which are simple, inexpensive, and readily available markers of systemic inflammation, may help in decision making precancerous pathologies of the cervix. Nevertheless, future studies with larger patient groups are essential to confirm the results of this preliminary study.

## Figures and Tables

**Table 1:** Age and hematological parameters of the study population

*N* = 110	
Age (years)	38 ± 10
White blood cell count (10^3^/*μ*L)	7.7 ± 2.3
Hemoglobin (g/dL)	12.7 ± 1.6
Red cell distribution width (%)	13.7 ± 2.1
Plateletcrit (%)	0.26 ± 0.7
Platelet count (*μ*L)	273 ± 74
Mean platelet volume (fL)	9.6 ± 1.4
Neutrophil to lymphocyte ratio	2.2 ± 0.9
Platelet to lymphocyte ratio	131 ± 57

**Table 2:** Comparison of age and blood parameters in patients with LSIL, HSIL, and cervix cancer and controls.

	Patients with LSIL	Patients with HSIL	Patients with cancer	Controls	Overall *p*
	*n* = 40	*n* = 40	*n* = 30	*n* = 70	
Age (years)	39 ± 9	35 ± 7	46 ± 11^a^	35 ± 11	<0.001
WBC count (10^3^/*µ*L)	7.7 ± 2.6	7.5 ± 1.9	7.3 ± 2.7	7.9 ± 1.8	0.650
Hemoglobin (g/dL)	13.2 ± 1.2	12.9 ± 1.3	12.2 ± 1.7^b^	12.4 ± 1.7	0.020
RDW (%)	13.5 ± 1.5	14.0 ± 1.9	14.4 ± 2.8	13.3 ± 1.9	0.093
Plateletcrit (%)	0.26 ± 0.1	0.26 ± 0.1	0.29 ± 0.1^c^	0.24 ± 0.1	0.010
Platelet count (*µ*L)	263 ± 68	257 ± 65	303 ± 92^d^	274 ± 70	0.058
MPV (fL)	9.9 ± 1.1	10.1 ± 1.0	10.0 ± 0.8	8.9 ± 1.6^e^	<0.001
NLR	2.2 ± 1.1	2.3 ± 1.0	2.7 ± 1.1^c^	2.0 ± 0.8	0.016
PLR	123 ± 46	123 ± 34	191 ± 83^a^	119 ± 45	<0.001

HSIL: high-grade squamous intraepithelial lesion, LSIL: low-grade squamous intraepithelial lesion, MPV: mean platelet volume, NLR: neutrophil-to-lymphocyte ratio, PLR: platelet-to-lymphocyte ratio, RDW: red cell distribution width, and WBC: white blood cell. ^a^*p* < 0.05 between group 3 and groups 1, 2, and 4. ^b^*p* < 0.05 between group 3 and group 1. ^c^*p* < 0.05 between group 3 and group 4. ^d^*p* < 0.05 between group 3 and group 2. ^e^*p* < 0.05 between controls and groups 1, 2, and 3.

**Table 3:** Correlation of selected laboratory measurements with the presence of cervical cancer.

	*r*	*p*
Mean platelet volume	0.119	0.110
Plateletcrit	0.176	0.062
Neutrophil-to-lymphocyte ratio	0.223	0.003
Platelet-to-lymphocyte ratio	0.452	<0.001

**Table 4:** Logistic regression analysis illustrating the predictive value of age and laboratory measurements on the presence of cervical cancer.

	OR	95% CI	*p* value
Age	1.075	1.020–1.132	0.007
WBC count	1.181	0.692–2.016	0.542
Plateletcrit	3.782	0.223–6.749	0.612
Hemoglobin level	0.816	0.613–1.085	0.163
MPV	1.669	0.983–2.831	0.089
NLR	1.643	1.009–3.142	0.047
PLR	1.032	1.003–1.062	0.029

MPV: mean platelet volume, NLR: neutrophil-to-lymphocyte ratio, PLR: platelet-to-lymphocyte ratio, and WBC: white blood cell.

**Table 5:** Linear regression analysis demonstrating the association of age and laboratory parameters with FIGO stages in patients with cervical cancer.

	Coefficients-*β*	95% CI	*p*
Age	0.005	−0.012–0.022	0.536
WBC count	−0.028	−0.119–0.063	0.530
Plateletcrit	−0.005	−0.114–0.105	0.932
Hemoglobin level	0.029	−0.221–0.279	0.813
MPV	1.007	−1.720–3.734	0.452
NLR	0.315	0.026–0.605	0.034
PLR	0.005	0.001–0.009	0.014

MPV: mean platelet volume, NLR: neutrophil-to-lymphocyte ratio, PLR: platelet-to-lymphocyte ratio, and WBC: white blood cell.

## Data Availability

The data used to support the findings of this study are available from the corresponding author upon request.
